# First application of three-dimensional designing total hip arthroplasty with long uncemented stem for fibrous dysplasia patients combined with hip joint osteoarthritis

**DOI:** 10.1186/s12891-019-2608-y

**Published:** 2019-05-17

**Authors:** Kai Yao, Li Min, Fan Tang, Minxun Lu, Yuqi Zhang, Jie Wang, Yong Zhou, Yi Luo, Wenli Zhang, Chongqi Tu

**Affiliations:** Department of Orthopedics, West China Hospital, Sichuan University, Guoxue Xiang No. 37, Chengdu, Sichuan 610041 People’s Republic of China

**Keywords:** Fibrous dysplasia, Osteoarthritis, 3D designing, Total hip arthroplasty, Long uncemented stem

## Abstract

**Background:**

In order to treat proximal femur fibrous dysplasia (FD) patients combined with hip joint osteoarthritis (OA), the three-dimensional (3D) designing osteotomy and implantation of femoral component was firstly used for deformity correction and total hip arthroplasty (THA). The purpose is to present the detailed design, perioperative management and evaluate short-term clinical outcomes of this novel therapeutic method.

**Method:**

A retrospective study was performed in twelve FD patients combined with hip joint OA who were treated in our hospital between July 2013 and April 2015. Seven patients received 3D designing combined osteotomy and THA, and the other five patients underwent 3D designing THA only.

**Results:**

All patients were followed-up with an average duration of 47 months (range, 35–56 months). There was no infection, dislocation, postoperative wound problems or mechanical failures. For the seven patients receiving 3D designing corrective osteotomy, the mean extremity lengthening was 2.8 (range, 1.5–4) cm. The average duration of bone union was 4.2 months. The average Harris Hip Score was improved from 46.08 (range, 13–67) points preoperatively to 93.72 (range, 83–100) points at the last follow-up. The average modified criteria of Guille was improved from 3.2 (range, 1–7) points preoperatively to 8.6 (range, 6–10) points at the last follow-up.

**Conclusions:**

The 3D designing THA with long uncemented stem, including 3D designing corrective osteotomy and implantation of long prosthesis stem, seems to be a reliable method for FD patients combined with hip joint OA. Through preoperative 3D design, corrective osteotomy and implantation of long prosthesis stem can be precise to re-store alignment, uttermost preserve host bone, obtain primary stem stability and provide necessary condition for long-term stem survival, finally leading to better limb function. Besides, perioperative management should be abided strictly for late stability. Nevertheless, the outcomes of long-term follow-up and larger cases are still required.

## Background

Fibrous dysplasia (FD) is a benign lesion characterized by the replacement of normal bone tissue with immature fibro-osseous tissue leading to widening and thinning of the cortex. It can occur in single bone (monostotic) or multiple bones (polyostotic) [1]. The proximal femur, which is the most common site, can gradually form progressive coxa vara and bowing deformity under the continuous mechanical stress and repeated fractures, and result in functional impairment and pain [2–4].

In a large proportion of these patients, secondary hip joint osteoarthritis (OA) can be caused by the abnormal mechanical alignment of the proximal femur [5, 6]. Besides, the severity of the deformity and the presence of FD lesions in the peri-articular bones (head of femur and acetabulum) were proven to be independently and closely associated with the development of secondary hip joint OA in these patients [7]. Although, the reported incidence of secondary hip joint OA in patients with proximal femur FD was 13% [7], with the disease progression, the final incidence will highly increase.

The surgical treatment for proximal femur FD combined with hip joint OA remains challenging due to the poor bone conditions, severe bone deformity, dysfunctional hip abductor muscle and non-standard implant choice. Recently, some literatures have focused on this problem, total hip arthroplasty (THA) has been accepted as an effective method [6]. The short uncemented stem or cement fixation were applied, however, the postoperative prosthesis loosing was severe, long-time fixation of femoral stem was unsatisfactory, and the number of cases was limited [6, 8, 9].

3D (three-dimensional) image technology has opened up a new era to this problem. In order to preserve the remaining femoral bone stock and attain a more precise, individual and durable prosthesis implantation, the 3D designing THA with long uncemented stem was firstly applied in our institution. This study attempts to present the detailed design procedures, perioperative management and evaluate short-term clinical outcomes of this novel therapeutic method.

## Patients and methods

### Patients

Twelve patients with proximal femur FD combined with hip joint OA underwent THA with long uncemented stem (solution revision femoral prosthesis (Johnson & Johnson (Shanghai) Medical Device Co., Ltd)) between July 2013 and April 2015 in our hospital. There were four males and eight females. The average age was 46.3 years (range, 31–65 years). The mean BMI (body mass index) was 22.47 (range, 19.05–26.99). All patients were diagnosed with FD combined with hip joint OA. Pathological fractures occurred in three patients when admission. Among them, there were ten patients with polyostotic disease and two patients with monostotic disease. The average duration of bone and joint symptoms was 22.75 years (range, 7–48 years) (Table 1).

**Table 1:** Patient characteristics and outcomes

Case	Gender/age at operation(year)	BMI	Type of lesion	Radiographic classification	Pathological fracture	OA	Duration of bone and joint symptoms (year)	operation	Length of follow-up (months)	Clinical course	Harris Scores (pre−/post-operative)
1	F/61	20.94	P	Type 5	No	Yes	48	Osteotomy + THA	56	Asymptomatic Osteotomy healed	20/83
2	F/39	19.05	P	Type 4	No	Yes	21	Osteotomy + THA	55	Asymptomatic Osteotomy healed	56/97
3	M/31	22.59	P	Type 5	Yes	Yes	22	Osteotomy + THA	53	Asymptomatic	13/96
4	M/47	26.99	M	Type 2	No	Yes	7	THA	51	Asymptomatic	63/100
5	F/32	19.47	P	Type 5	No	Yes	18	Osteotomy + THA	49	Asymptomatic Osteotomy healed	61/97
6	M/38	24.51	P	Type 4	No	Yes	23	Osteotomy + THA	48	Asymptomatic Osteotomy healed	32/93
7	F/65	22.60	P	Type 3	Yes	Yes	19	THA	47	Asymptomatic	16/83
8	F/52	21.23	M	Type 2	No	Yes	12	THA	46	Asymptomatic	45/96
9	F/45	23.50	P	Type 5	Yes	Yes	35	Osteotomy + THA	45	Asymptomatic Osteotomy healed	58/83
10	M/58	22.99	P	Type 4	No	Yes	33	Osteotomy + THA	42	Asymptomatic Osteotomy healed	58/97
11	F/40	20.96	P	Type 3	No	Yes	13	THA	37	Asymptomatic	67/100
12	F/48	24.84	P	Type 3	No	Yes	22	THA	35	Asymptomatic	64/100
Mean	−/46.3	22.47					22.75		47		46.08/93.72

Notes: Harris Scores range: 0–100. BMI: body mass index; P: polyostotic disease; M: monostotic disease; OA: osteoarthritis; THA: total hip arthroplasty

All patients underwent X-ray, three-dimensional computed tomography (3D-CT) and bone scintigraphy. According to our institution’s radiographic classification of FD in the proximal femur [10], there were two type 2 patients, three type 3 patients, three type 4 patients, and four type 5 patients (Table 1).

### Preoperative design

**Fig. 1:**
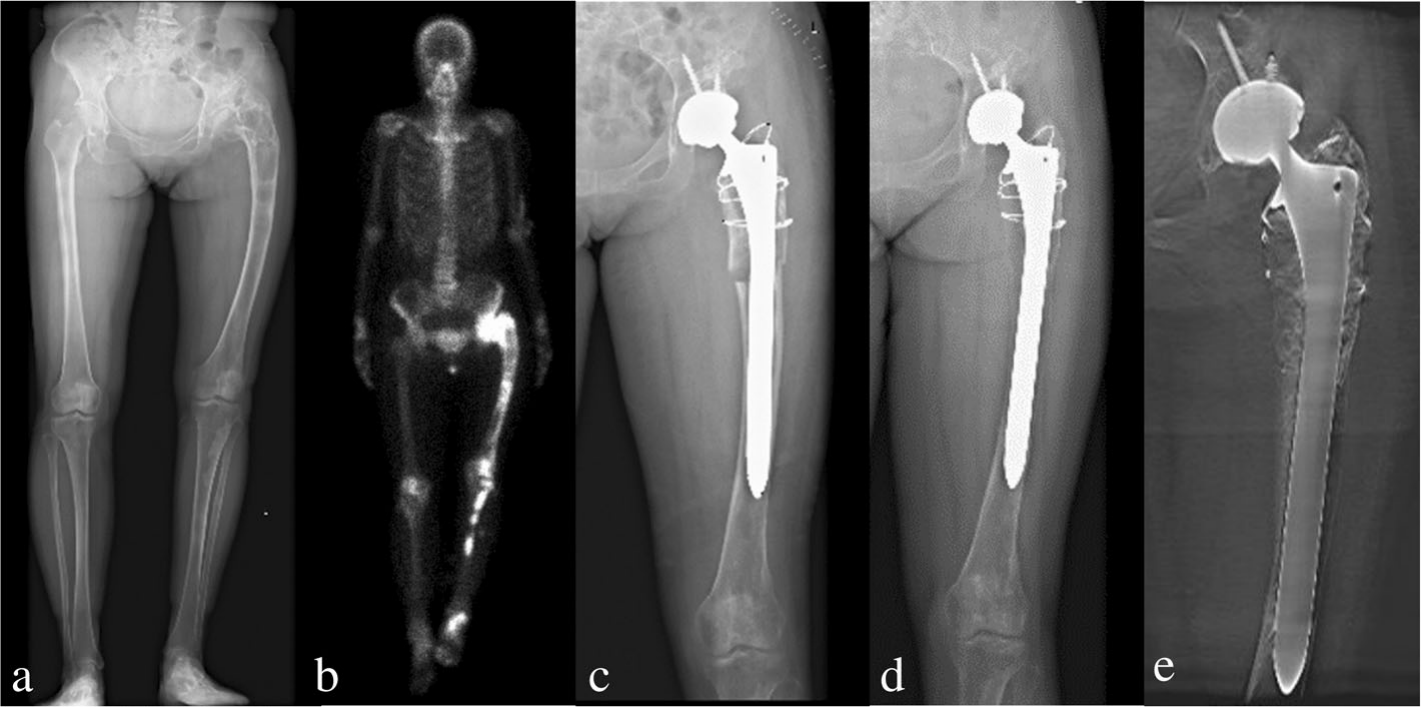
Preoperative radiography (**a**), bone scintigraphy (**b**), postoperative radiography (**c**), and at the 18 months follow-up of radiography (**d**) and T-SMART image (**e**) in patient 1

**Fig. 2:**
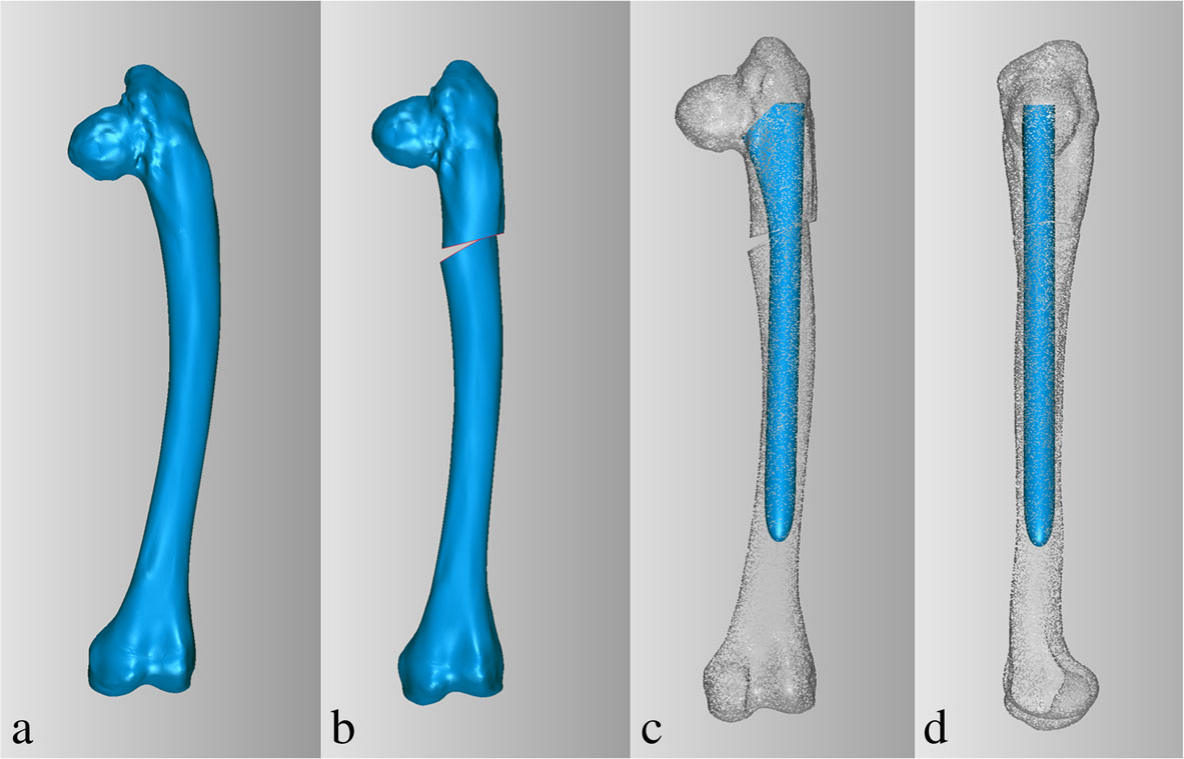
3D computer models of the femur (**a**), 3D designing corrective osteotomy (**b**), 3D designing implantation of long prosthesis stem (**c, d**) in patient 1

**Fig. 3:**
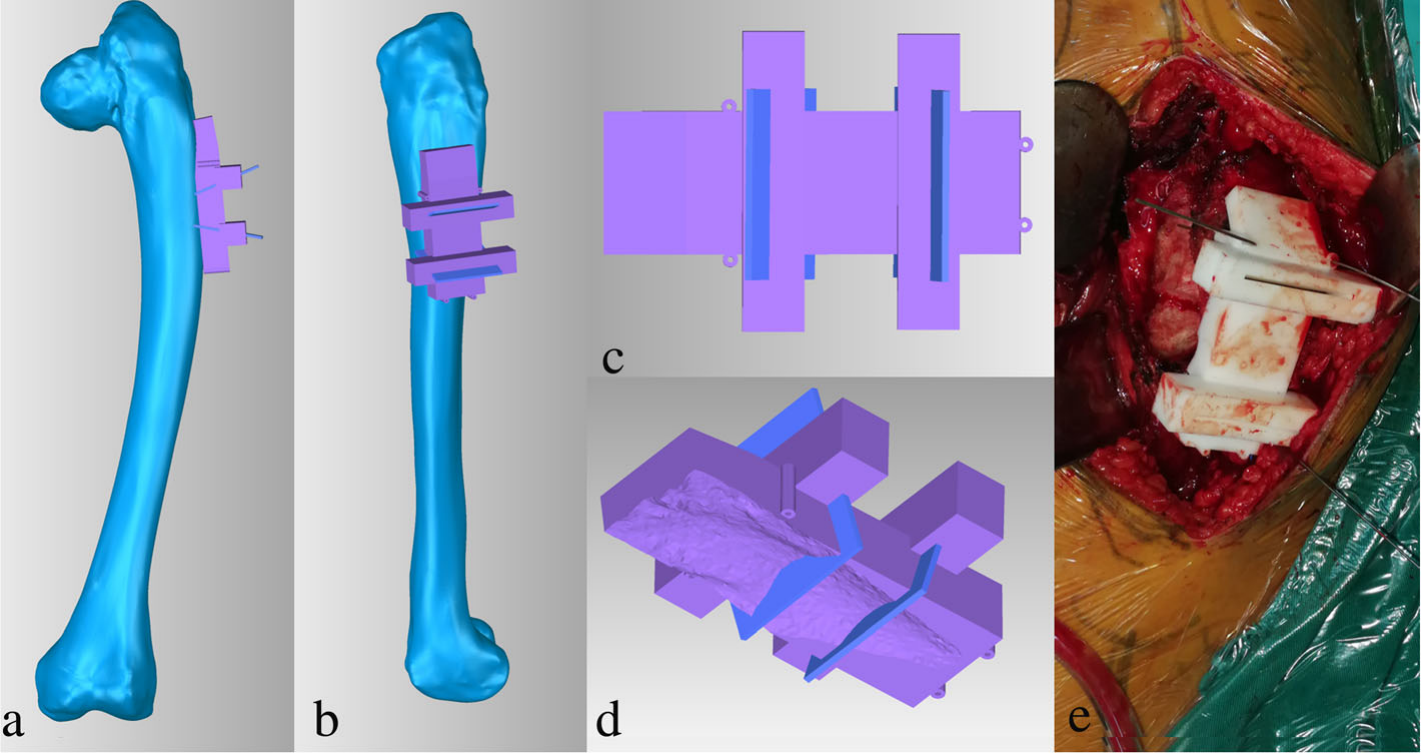
Customized osteotomy guides was designed according to the osteotomy method determined by 3D design (**a, b**),the osteotomy guides have osteotomy guide auxiliary groove, an internal surface completely matched with the bone surface, a position mark and four kirschner wire fixation holes (**c, d**),intraoperative photo of applying osteotomy guide (**e**)

For preoperative planning, simulation of osteotomy and implantation of prosthesis stem in 3D reconstructive model were performed. Firstly, the data from the 3D-CT scan was imported into Mimics V17.0 Software (Materialise Corp. Belgium), and 3D computer femoral models for these patients were built. On 3D reconstructive model, the mechanical alignment of the femur could be determined, and the osteotomy requirements including position, size and angle could be simulated. Based on the 3D reconstructive model after corrective osteotomy, the femoral morphology and mechanical alignment could be evaluated. Through simulating the implantation of prosthesis stem, the size and diameter of the prosthesis stem which matched with the medullary cavity, the optimal insertion points and direction of implantation were determined. The osteotomy process was very important in the surgical procedure, which directly affected the effect of correction and prosthesis implantation. Furthermore, the long-term prothesis stability and postoperative function were also affected. In order to improve the accuracy of osteotomy, customized osteotomy guides were made by 3D designing after the osteotomy method was determined (Figs. 1, 2 and 3).

### Surgical technique

All of the operations were performed by a senior surgeon (Tu Chongqi). A lateral position was used. A standard surgical approach though hip posterior lateral incision was routinely conducted to expose the acetabulum and proximal femur. All cases underwent the following procedures. These procedures included four steps, corrective osteotomy, curettage of the lesion totally, massive impaction allograft, and implantation of acetabular cup and long uncemented stem. Firstly, corrective osteotomy was performed assisted by customized osteotomy guides in this study, which guaranteed the accuracy of the operation. Secondly, the FD lesions, which would affect bone and prosthesis bone integration, should be completely removed. Different type of bone curettes should be used to remove the lesions because the lesions cavities were irregular. Rapid and complete scrape to normal bone or gelatin sponge compression were used to control intramedullary bleeding. Thirdly, massive impaction allograft should be obtained to improve the ability of bone integration between prosthesis and bone. The cavities of the proximal femur and periacetabular lesions after curettage were irregular. The bone ridge of the medullary wall was helpful to match the prosthesis stem and improve the initial stability of the prosthesis. The cavities in the medullary wall and periacetabular should be grafted by allogeneic cancellous bone to improve the contact between bone and prosthesis, increase bone integration and improve long-term stability. The smaller prosthesis intramedullary stem could be inserted into the medullary cavity firstly, and then bone grafting was grafted around the intramedullary stem to partially fill the cavity. Allogenic cancellous bone should be prepared to less than 2 mm in diameter to increase the amount of bone graft. Finally, after massive impaction allograft, the suitable size prosthesis femoral stem, which determined by preoperative 3D design, was pressed into the femoral intramedullary cavity one-time.

In our series, three type 4 and four type 5 patients with varus deformity in the proximal femoral shaft received 3D designing single level valgus osteotomy.

### Postoperative management

The postoperative management was determined by the primary stability of the stem. The patients with good primary stability and bone condition were allowed 50% weight-bearing exercise after 6 weeks no weight-bearing exercise. Otherwise, no weight-bearing exercise was allowed until 6 weeks postoperatively. The subsequent program for increasing weight-bearing exercise was determined by the clinically and radiography follow-up results.

### Follow up

**Fig. 4:**
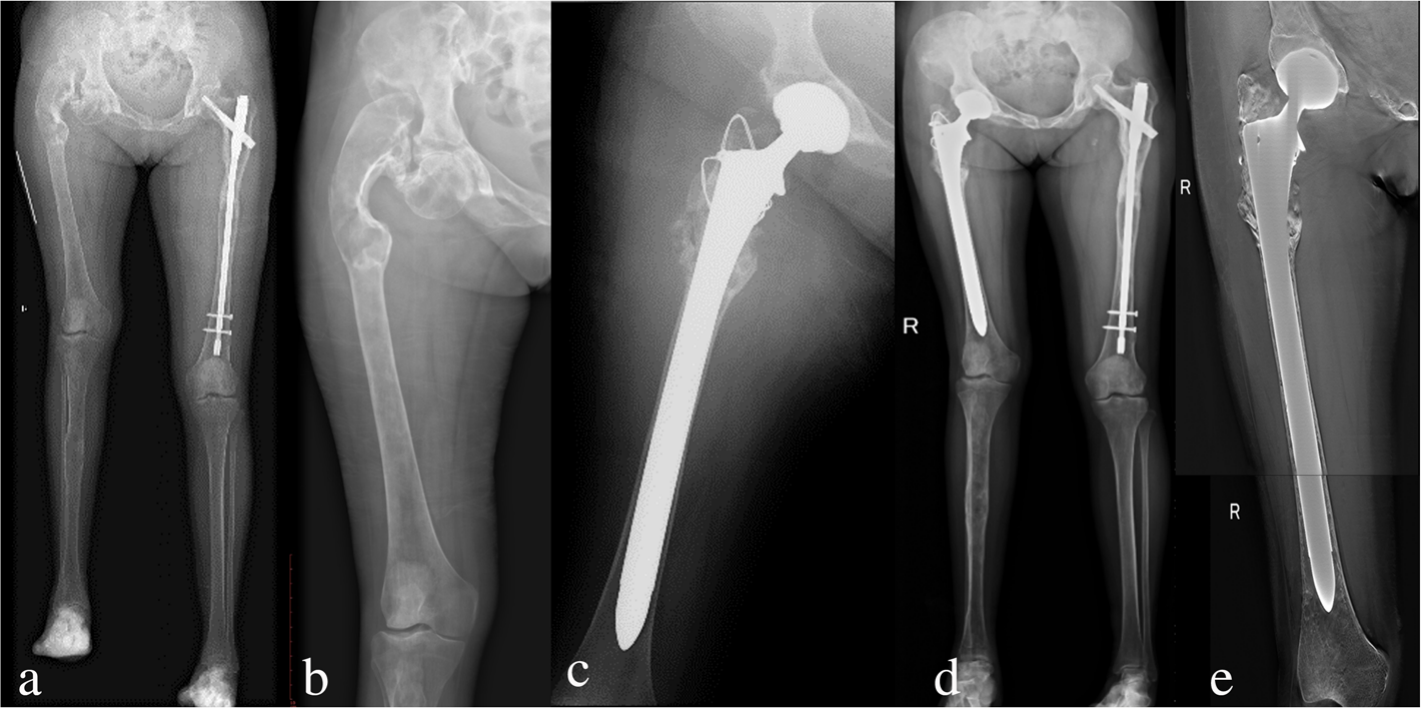
Preoperative radiography (**a, b**), postoperative radiography (**c**), and at the 36 months follow-up of radiography (**d**) and T-SMART image (**e**) in patient 9

All patients were followed-up clinically and radiologically. Information obtained included: union of osteotomy, pain relief and limb length discrepancy were compared before and after surgery. The final function of the hip was evaluated by the Harris Hip Score [11] and the modified criteria described by Guille et al. [12]. Radiographic stability was confirmed by the implant osteointegration and osteotomy healing on the T-SMART (tomosynthesis-shimadzu metal artefact reduction technology) images (Figs. 1 and 4).

### Statistical analysis

The normality of the continuous data was checked by the one sample Kolmogorov-Smirnov test. Normally distributed parameters were assessed by independent-samples t-test, and nonnormally distributed parameters were assessed by the Mann-Whitney U test. A *p* < 0.05 was considered statistically significant.

## Results

In our series, seven patients received 3D designing combined osteotomy and THA, and the other five patients underwent 3D designing THA. The mean surgical time was 150 (range, 125–190) min. The average amount of intraoperative bleeding was 730 (range, 550–1350) ml. There was no infection, dislocation, thromboembolism, postoperative wound problems or mechanical failures.

The average follow-up was 47 (range, 35–56) months (Table 1). All patients had normal functions of the knee joint, ankle joint and foot, and satisfactory function of hip joint. At the last follow-up, 7 cases were painless, 5 patients had significant relief of pain. All cases were able to walk, even though 3 cases walked with the aid of cane during a long walk. Only one patient got residual deformity of lower limbs because of the severe bone condition preoperatively, but the function outcome for this patient was satisfactory.

**Fig. 5:**
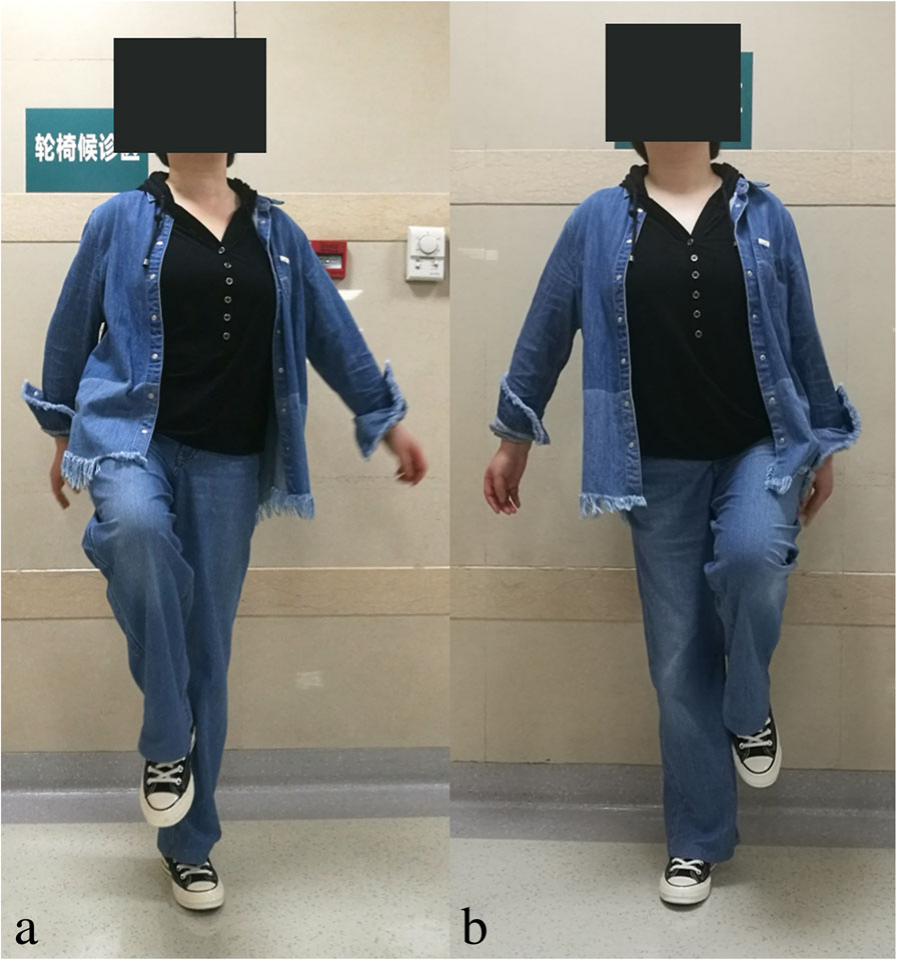
Functional results (**a, b**) of the left hip in patient 9 at the follow-up of 36 months

**Fig. 6:**
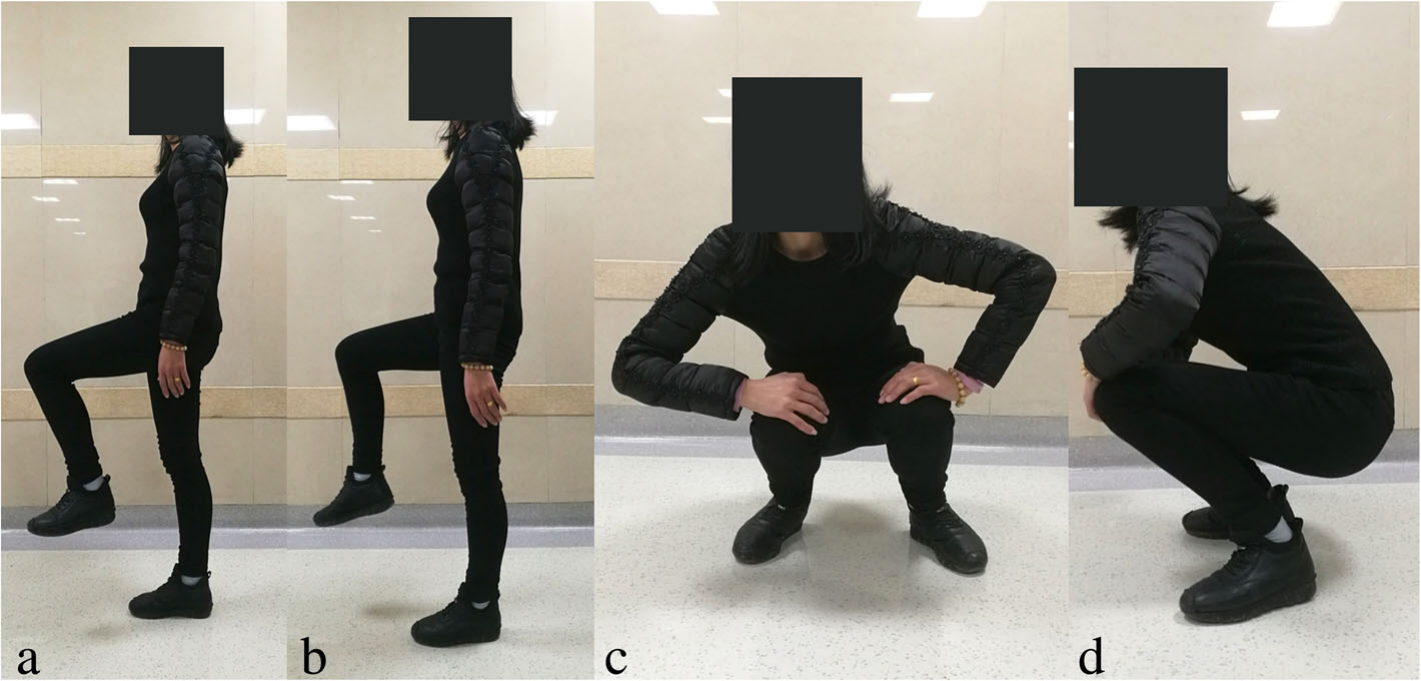
Functional results (**a, b, c, d**) of the right hip in patient 5 at the follow-up of 42 months

The average Harris Hip Score was improved from 46.08 (range, 13–67) points preoperatively to 93.72 (range, 83–100) points at the last follow-up (Table 1). The average modified criteria of Guille was improved from 3.2 (range, 1–7) points preoperatively to 8.6 (range, 6–10) points at the last follow-up. Results were excellent in 7 patients (Figs. 5 and 6), good in 4 patients, fair in 1 patient, and poor in 0 patients. The details of clinical outcomes were shown as Table 2.

**Table 2:** comparison of preoperative and postoperative hip function according to modified criteria of Guille

Categories	Unsatisfactory		Average		Satisfactory	
	Preoperative	Postoperative	Preoperative	Postoperative	Preoperative	Postoperative
Pain	6	0	5	5	1	7
Hip motion	7	0	3	3	2	9
Limping	7	0	4	4	1	8
Activities of daily living	6	0	4	3	2	9
Social activities	5	0	4	2	3	10

Notes: 12 femora were evaluated by five different categories. Hip function: unsatisfactory scored 0 points, average scored 1 point, satisfactory scored 2 points. In a case with a maximum score of 10 points, ≥9 points was identified as excellent, 7 or 8 points as good, 5 or 6 points as fair, and < 5 points as poor

**Fig. 7:**
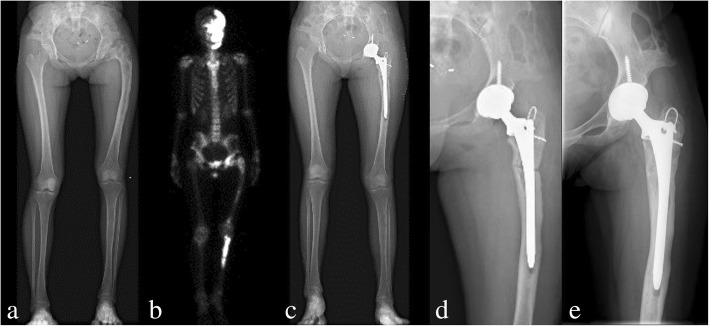
Preoperative radiography (**a**), bone scintigraphy (**b**), postoperative radiography (**c, d**), and radiography at the follow-up of 42 months (**e**) in patient 5

All of the patients were divided into two groups according to whether or not received osteotomy, including seven patients received 3D designing combined osteotomy and THA and five patients underwent 3D designing THA only. For the seven patients received 3D designing valgus osteotomy, the limb-length discrepancy was corrected from 3.8 (range, 1.5–7) cm preoperatively to 1.0 (range, 0–3) cm postoperatively. The mean extremity lengthening was 2.8 (range, 1.5–4) cm (Figs. 1, 4 and 7). No postoperative neurovascular complications or dysfunction of muscle was found owing to the lengthening of the lower limb. The bone union was achieved in all patients, and the average healing time was 4.2 (range, 3–6) months. The details of Harris Hip Score were shown as Table 3. Significant differences were only detected in limp (*p* = 0.020) between the two groups. But there were no significant differences in pain (*p* = 0.930), distance walked (*p* = 0.763), support (*p* = 0.763), stair climbing (*p* = 0.763), sitting (*p* = 1.000), shoes and socks (*p* = 1.000), transportation (*p* = 1.000), deformity (*p* = 0.424), range of motion (*p* = 0.763), and Harris Hip Scores (*p* = 0.402) between the two groups (Table 3).

**Table 3:** Postoperative clinical outcome evaluated by Harris hip score

Case	Pain	Distance walked	Support	Limp	Stair climbing	Sitting	Shoes and socks	Transportation	Deformity	Range of motion	Harris scores
1	40	8	7	8	2	5	4	1	4	4	83
2	44	11	11	8	4	5	4	1	4	5	97
3	40	11	11	11	4	5	4	1	4	5	96
4	44	11	11	11	4	5	4	1	4	5	100
5	44	11	11	8	4	5	4	1	4	5	97
6	40	11	11	8	4	5	4	1	4	5	93
7	40	8	7	8	2	5	4	1	4	4	83
8	40	11	11	11	4	5	4	1	4	5	96
9	44	8	7	8	2	5	4	1	0	4	83
10	44	11	11	8	4	5	4	1	4	5	97
11	44	11	11	11	4	5	4	1	4	5	100
12	44	11	11	11	4	5	4	1	4	5	100

Notes: Harris Scores range: 0–100

## Discussion

For FD combined with hip joint OA patients, especially who have severe proximal femur deformity, corrective osteotomy and THA are both needed. Corrective osteotomy can solve local deformity and re-establish mechanical alignment of the lower extremity [1]. THA can relieve pain and improve the limb function [6]. Some previous publications have demonstrated the value of application of computer-assisted technology, robotics-assisted technology and rapid prototype model in complex hip surgery. One review has summarized computer-assisted technology, robotics-assisted technology can help surgeons perform accurately to achieve the clinical objectives in complicated THA [13]. In 2013, Won et al. reported that the pelvic rapid prototype model was used in 21 complex hips to simulate the implantation of the acetabular prosthesis preoperatively, the implantation of acetabular component was successful and 80.9% of the used acetabular component was within 2 mm of the predicted size [14]. In 2016, Inaba used 3D preoperative planning and intraoperative navigation for rotational acetabular osteotomy in acetabular dysplasia patients, the rotational acetabular osteotomy could be performed more accurate and safer [15]. But the 3D preoperative design is still rare applied in THA with severe proximal femoral deformity. In order to improve the perioperative accuracy rate of corrective osteotomy and THA, 3D preoperative design was firstly used in FD combined with hip joint OA patients. This study demonstrated the details of 3D preoperative design and perioperative management, and the short-term outcomes of THA with long uncemented stem for this kind of patients.

With the progression of proximal femur FD, coxa vara deformity is inevitable. For this deformity, valgus osteotomy can improve the limb function and prevent fractures via re-establishing neck-shaft angle and reconstructing the lower limb strength line [16]. The ways of valgus osteotomy are based on the deformity classification. Ippolito proposed a classification method including six types in 2014 [17]. However, Ippolito’s classification has a major limitation, which is the surgeons cannot determine osteotomy site or internal fixation method according to this classification. In 2015, our institution reported a novel method of radiographic classification including five types according to three parameters: neck-shaft angle, varus deformity of the proximal femoral shaft, and reduction in proximal femoral strength [10]. In our series, according to our institution’s classification, there were two type 2 patients, three type 3 patients, three type 4 patients, and four type 5 patients. Femoral valgus osteotomy was done in the three type 4 and four type 5 patients, who had varus deformity in the proximal femur shaft. Nevertheless, the current classifications, including ours, only evaluate the femoral deformity but not the injury degree of the ipsilateral hip joint [10, 17]. Moreover, valgus osteotomy can lengthen the lower extremity to some extent. Previous researches demonstrated that corrective osteotomy can lengthen extremity by 2.3–3.4 cm on average [16, 18]. In our study, the lower limb has been lengthened by an average of 2.8 (range, 1.5–4) cm.

Current classifications of proximal femur FD are only based on the images of X-ray and CT, but not a multi-dimensional evaluation [10, 17]. To our best knowledge, there is no report using 3D designing femoral reconstruction to guide the operation. Additionally, the proximal femur medullary cavity is varied in diameter and the bone condition is poor for FD patients, the implantation of prosthesis stem is challenging. But it is still very rare to pay attention to the bone condition of the affected proximal femur, especially the surface of medullary cavity. The 3D reconstructive images can fully and precisely evaluate the patients’ femoral deformity sites, affected bone length, abnormal mechanical alignment, bone condition, and multi-level diameter of the medullary cavity. Our results revealed that 3D designing osteotomy did not reduce the hip joint function when comparing the patients only receiving THA and the patients receiving osteotomy and THA. The preoperative 3D design can not only make better plan of the osteotomy but also simulate the feasibility of implanting long prosthesis stem more accurately. During this procedure, we can confirm the length and diameter of the prosthesis stem before surgery, so that the surgical difficulties can be sharply decreased, and the mean operating time and the average amount of intraoperative bleeding can be controlled well.

Primary stability and long-term survival are the most important factors to consider when choose the prosthesis stem, especially for these patients with bone deformity and poor bone condition. Firstly, uncemented stem was chosen in our series. The reasons has been discussed that the affected bone characterized with the wide and irregular medullary cavity and thin cortex, is not strong enough to support the cement-bone interface [19, 20]. Additionally, due to the relatively massive intramedullary bleeding, uncontrollable bleeding will undoubtedly influence the cement-bone interface during cemented stem implantation [6]. What’s more, for patients who require femoral osteotomy, the cement fixation may affect osteotomy healing. Secondly, long stem should be applied. Sierra reported that proximal femur FD patients who received short uncemented stem fixation showed a high rate (60%) of early aseptic loosening, and long stem prosthesis was highly recommended for proximal femur FD patients combined with hip joint OA. Besides, they suggested that the stems length should exceed the lesion length by at least 2 femoral canal diameters to decrease the risk of postoperative fractures [6]. Some data demonstrated that besides long stem, increasing bone mass by cortical bone or compressive bone grafting and custom-made megaprosthesis can also improve the integral strength of the proximal femur [21]. The same theory has been accepted during femoral revision surgery after ordinary THA [22, 23]. What’s more, in some rarely extreme cases, if the usual size of the femoral stem (solution revision femoral prosthesis) was not appropriate for the patient, the customized femoral stem could be produced assisted by 3D design preoperatively.

The surgical skill of the stem implantation is another crucial point worthy of notice. First, precision osteotomy assisted by 3D designing customized osteotomy guides should be performed and micropendulum saw was required to be used to reduce bone loss and cortical injury. Second, The lesion required to be removed thoroughly before reaming, which would prevent poor osteointegration after uncemented fixation [6]. Third, the femoral cavity needed to be reamed before the implantation of long stem. 3D designing preoperative plan and intraoperative X-ray fluoroscopy played a very important role in the precise reaming of the bone cavity and preventing cortical bone damage. Fourth, in order to increase initial stability, the diameter of reaming needed to be slight (0.5 mm) smaller than the diameter of the stem because of the flexibility of the cortical bone with FD. Fifth, during stem implantation, the anteversion angle of the stem should be pay attention to, because the anteversion angle of the host proximal femur is always less than normal. Sixth, the procedure of wire binding was usually used to re-establish abductor muscle and prevent intraoperative periprosthetic fractures [24]. During osteotomy and prosthesis implantation, periosteal should be protected to promote bone healing. Finally, massive impaction of cancellous allograft should be used before the implantation of acetabular cup and prosthesis stem. Massive impaction allograft is the critical procedure to improve the potential of osteointegration and to prevent uncemented implant loosening [16, 25, 26].

It should be noted that this is a retrospective and short-term follow-up study related to 3D designing THA with long uncemented stem for FD patients combined with hip joint OA. Additionally, this study included a limited number of patients. Nevertheless, this study still demonstrates the clinical value and potential of 3D designing osteotomy and implantation of long uncemented stem for FD patients combined with hip joint OA.

## Conclusion

The 3D designing THA with long uncemented stem seems to be a reliable method for FD patients combined with hip joint OA. To our best knowledge, this study includes the largest number of patients using long uncemented stem and is the first application of 3D design technology for osteotomy and stem implantation currently. The 3D designing corrective osteotomy can re-build the normal physiological structure of the affected bone accurately and improve the lower limb function. The 3D designing implantation of long prosthesis stem is more precise, contributes to primary stability, and provides necessary condition for long-term survival. Moreover, perioperative management should be carefully paid attention to. Nevertheless, the long-term outcomes and larger cases results are still required.

## Abbreviations

3D: three-dimensional
FD: fibrous dysplasia
OA: osteoarthritis
THA: total hip arthroplasty
T-SMART: tomosynthesis-shimadzu metal artefact reduction technology

## Authors’ information

Department of Orthopedics, West China Hospital, Sichuan University, Guoxue Xiang #37, Chengdu, Sichuan 610041, People’s Republic of China.
